# AI-Enabled Flexible Sensing Ecosystems for Parkinson’s Disease: Advancing Digital Biomarkers and Closed-Loop Interventions

**DOI:** 10.3390/s26072071

**Published:** 2026-03-26

**Authors:** Jiadong Jin, Yongchang Jiang, Yukai Zhou, Wenkai Zhu, Jiangbo Hua, Wen Cheng, Yi Shi, Lijia Pan

**Affiliations:** 1 Collaborative Innovation Center of Advanced Microstructures, Jiangsu Provincial Key Laboratory of Photonic and Electronic Materials, School of Electronic Science and Engineering, Nanjing University, Nanjing 210093, China; 522025230032@smail.nju.edu.cn (J.J.);; 2 School of Integrated Circuits, Nanjing University, Suzhou 215163, China

**Keywords:** Parkinson’s disease, flexible sensors, artificial intelligence, deep learning, freezing of gait, digital biomarkers

## Abstract

Effective Parkinson’s disease (PD) management is hindered by the intermittent nature of clinical snapshots and the discomfort of rigid monitoring hardware. This review critically evaluates the synergy between flexible bioelectronics and artificial intelligence (AI) for continuous remote monitoring. Our analysis reveals that while material innovations have achieved milligram-level sensitivity, a significant ‘translational gap’ persists due to limited validation in real-world environments and small cohort sizes. We conclude that multimodal fusion architectures are essential for accurately mapping digital biomarkers to clinical gold standards such as MDS-UPDRS. By leveraging edge AI for privacy and closed-loop feedback for intervention, this integration facilitates the transition from reactive clinical visits to proactive, personalized digital home-care ecosystems.

## 1. Introduction

### 1.1. Clinical Background: Symptom Volatility and the Urgency of Continuous Home Monitoring

PD is currently the fastest-growing neurodegenerative disorder worldwide. According to recent statistics, the global prevalence of PD has reached 6.2 million and is projected to increase to 14.2 million by 2040 [1]. The pathological hallmark of PD is the progressive loss of dopaminergic neurons in the substantia nigra of the midbrain, leading to classic motor symptoms such as bradykinesia, resting tremor, and muscle rigidity [2,3]. These symptoms severely affect patients’ professional and daily living abilities, ultimately reducing their quality of life [4,5,6]. Many people misinterpret these motor manifestations as natural consequences of aging, leading to low clinical consultation rates. As the disease progresses, more than half of patients develop Freezing of Gait (FoG)—a highly traumatic symptom that serves as a critical driver of falls, disability, and a precipitous decline in overall well-being [7].

However, existing clinical management frameworks face serious challenges in achieving precision care. First, the accuracy of early diagnosis ranges only from 58% to 85%, leaving a substantial number of symptomatic individuals undiagnosed [8]. Second, the clinical quantification of FoG is exceptionally complex. Due to the transient, unpredictable nature of episodes and their high environmental dependency, patients often struggle to accurately articulate the classic subjective sensation of being “glued to the floor” [9]. In controlled outpatient settings, physicians find it extremely difficult to capture these stochastic freezing events due to the absence of provocative triggers such as narrow spaces or turns. Consequently, “snapshot” assessments based on the Movement Disorder Society-Sponsored Revision of the Unified Parkinson’s Disease Rating Scale (MDS-UPDRS) fail to objectively reconstruct real-world symptom fluctuations, such as the “on-off phenomenon” that occurs at the end of medication cycles [10].

Furthermore, although rigid sensors have demonstrated high screening accuracies, their substantial bulk, poor comfort, and susceptibility to motion artifacts severely constrain long-term patient adherence [8]. Simultaneously, continuous cueing interventions are prone to inducing a “habituation” effect in the brain, causing assistive efficacy to attenuate over time. The inherent contradiction between clinical imperatives and hardware limitations has catalyzed the advancement of flexible bioelectronics. Integrating high-performance flexible sensing mechanisms with edge AI algorithms (e.g., embedded machine learning and lightweight deep learning) to construct skin-conformant monitoring systems for real-time, objective quantification has become an inevitable trajectory for the precision diagnosis and treatment of Parkinson’s disease [11,12].

### 1.2. Research Status and Technical Evolution: From Clinical Scales to Flexible Sensing

The development of monitoring technology for PD has undergone a profound evolution from “subjective to objective” and from “intermittent to continuous.” This shift in technical pathways is essential for defining the research positioning of flexible sensors [13]. Historically, the diagnosis and staging of PD have relied heavily on the gold-standard MDS-UPDRS, which essentially serves as an “intermittent snapshot” based on clinician visual observations and patient self-reports [14]. The early 21st century witnessed a digital transformation, exemplified by the WATCH-PD study, in which researchers utilized Inertial Measurement Units (IMUs) integrated into smartphones and smartwatches to capture kinematic data, marking the formal entry of PD management into an era of objective quantification. However, traditional rigid wearable technologies face insurmountable bottlenecks in real-world applications:

- Risk of clinical underdiagnosis: Due to the transient and unpredictable nature of symptoms such as FoG, short-term assessments in controlled clinical environments fail to capture stochastic events in home settings [15,16].
- Adherence and social stigma: Elderly patients often experience psychological stress when wearing bulky, conspicuous devices, hindering long-term monitoring adherence [17,18,19,20].
- Signal distortion: The significant mismatch in Young’s modulus between rigid materials and human skin makes devices highly susceptible to mechanical displacement and signal artifacts during motion [21,22].

To overcome these limitations, PD monitoring is shifting toward a “flexible” paradigm. By introducing Kirigami structures, ultrathin elastic substrates and other advanced strategies to achieve skin-conformity, noise induced by interfacial sliding is eliminated at the physical level, enabling the high-fidelity capture of Force Myography (FMG) and Center of Pressure (COP) signals [23]. Furthermore, material innovations such as eutectic gels provide the hardware foundation for constructing long-endurance, closed-loop intervention systems [24]. This transformation is not merely a change in form factor but a profound integration of sensing and computing: by integrating lightweight edge deep learning algorithms, flexible patches can perform feature extraction and risk warning directly on-device, achieving rapid-response, privacy-preserving, and “imperceptible” full-time health management [25].

### 1.3. Synergy Between Flexible Sensing and AI: A New Paradigm for Digital Biomarkers

**Figure 1:**
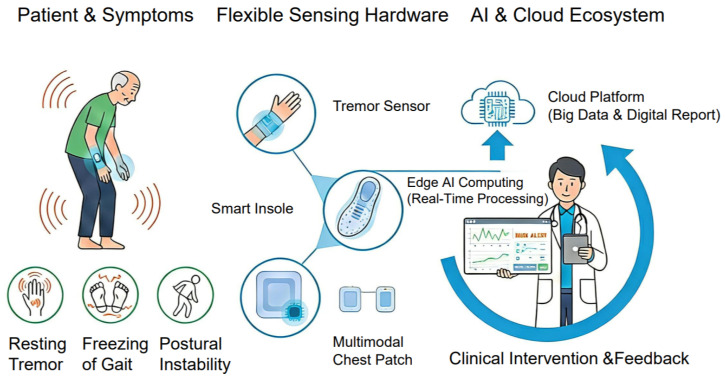
Conceptual framework of an AI-enabled flexible sensing ecosystem for closed-loop PD management. It illustrates the integration of symptom capture, flexible hardware sensing, and AI-driven clinical decision-making.

The overall conceptual framework of the AI-enabled flexible sensing ecosystem for closed-loop PD management is depicted in Figure 1, illustrating the seamless integration of symptom capture, flexible hardware sensing, and AI-driven clinical decision-making. The convergence of flexible bioelectronics and AI is catalyzing a paradigm shift in the management of PD, transitioning from intermittent clinical observations to the era of “Digital Biomarkers.” Digital biomarkers refer to objective, quantifiable physiological and behavioral data captured by digital devices to explain, influence, or assess health-related outcomes. In the context of PD, these biomarkers—ranging from tremor frequency and gait symmetry to postural stability—provide high-dimensional and continuous insights into the disease’s progression that traditional scales cannot capture.

The essence of this technological evolution lies in the deep synergy between hardware and software. High-performance flexible sensors act as the “sensory nerves” of the system, leveraging their skin-conformity and high sensitivity to capture raw, high-fidelity mechanical and electrophysiological signals. However, these raw data streams are often coupled with non-linear artifacts and environmental noise. AI serves as the “digital brain,” employing advanced signal processing and deep learning architectures to deconvolve complex data, extract pathological features, and map them to clinical semantics (e.g., MDS-UPDRS scores). This “sensing-computing” integration not only compensates for the physical limitations of flexible materials, such as hysteresis and signal drift, but also enables real-time, on-device decision-making, ensuring both rapid response and patient data privacy.

### 1.4. Core Contributions of This Review

While existing reviews often focus solely on hardware materials or algorithmic accuracy in isolation, this paper uniquely contributes to the field by providing a full-chain synthesis that spans from bio-inspired sensing mechanisms to advanced artificial general intelligence(AGI)-driven clinical decision-making. By providing a structured comparative analysis of diverse sensing mechanisms developed in recent years, we critically evaluate their technical advantages, such as sensitivity and response speed, against inherent trade-offs including hysteresis, signal drift, and environmental instability. Furthermore, this review explicitly identifies the translational barrier between laboratory proof-of-concept prototypes and real-world clinical deployment by discussing critical challenges in cohort scalability, validation environments, and regulatory compliance. Finally, we systematically explore the evolution of AI-driven analytical pipelines, highlighting the transition from traditional feature engineering to end-to-end deep learning architectures and autonomous closed-loop intervention frameworks tailored for personalized Parkinson’s care.

The remainder of this paper is organized as follows: Section 2 examines the physical foundations and material innovations of flexible sensing mechanisms tailored for PD symptoms. Section 3 focuses on the evolution of AI algorithms, from feature engineering to lightweight deep learning architectures for edge-side prediction. Section 4 discusses the current landscape of clinical applications, multimodal integration, and the system-level challenges toward real-world deployment. Finally, Section 5 summarizes the core achievements and offers a perspective on future trajectories in the field.

### 1.5. Literature Search Strategy and Inclusion Criteria

To ensure a rigorous and comprehensive synthesis of the field, a structured literature search was conducted across multiple electronic databases, including PubMed, Web of Science, and IEEE Xplore, covering the period from 2015 to 2026. The search utilized keyword combinations such as “Parkinson’s disease,” “flexible sensors,” “artificial intelligence,” “digital biomarkers,” “motor symptoms,” and “closed-loop interventions”.

As this manuscript is presented as a narrative review, the inclusion criteria focused on peer-reviewed studies that:

- demonstrated innovative flexible sensing mechanisms for PD symptoms;
- integrated AI-driven analytical frameworks for real-time or offline monitoring;
- provided clinical validation or discussed technical maturity (TRL) to distinguish between proof-of-concept and clinical-ready systems.

Approximately 180 articles were initially screened, with the final selection emphasizing works that bridge the “Valley of Death” between laboratory prototypes and real-world clinical implementation.

## 2. From PD Clinical Symptoms to Flexible Sensing Mechanisms

### 2.1. Core Motor Symptoms of PD and Sensor Performance Requirements

In the clinical management of PD, precisely capturing the dynamic fluctuations of motor symptoms is a fundamental prerequisite for optimizing treatment regimens. Flexible sensing systems must establish differentiated performance metric supports tailored to various core motor biomarkers of PD.

For resting tremor, the most characteristic symptom of PD, sensing systems require exceptionally high frequency resolution and response sensitivity. Since tremor frequencies typically range between 4 and 6 Hz, and micro-tremors in early stages generate minute mechanical displacements, sensors must possess milligram-level detection limits [26]. Guo et al. demonstrated the potential of flexible devices for capturing weak tremor waveforms using a bio-inspired micro-crack structure with a detection limit of 25 mg [27]. To ensure waveform reproduction without phase distortion, the electret sensor proposed by Xie et al. improved the response speed to 2.5 ms, meeting the hardware requirements for real-time quantification of high-frequency tremors [23].

Regarding the monitoring of bradykinesia and gait abnormalities, performance requirements shift toward linear response within a wide dynamic range and multimodal data fusion. Samarentsis et al. pointed out that plantar pressure monitoring systems need to cover a dynamic range up to 872.4 kPa while maintaining a linear correlation ($R^{2}$) above 0.993 to precisely segment gait phases such as heel-strike and toe-off [28]. Zhang et al. further emphasized that a single stream of kinetic data is insufficient to fully explain the neural mechanisms of bradykinesia [29]. By integrating IMUs with sEMG sensors, dual validation of muscle strength levels and movement amplitudes can be achieved, reaching a clinical consistency (Kappa value) exceeding 0.833 [30].

As the primary trigger for fall risks in PD patients, the monitoring focus of FoG has evolved toward “pre-episode prediction.” Research by Moore et al. showed that FoG episodes are often preceded by electrophysiological fluctuations caused by muscle cocontraction [31]. Capturing these precursor signals using flexible surface electromyography (sEMG) suits enables earlier risk warnings compared to traditional accelerometers. Furthermore, the shallow multi-view recognition network proposed by Chen et al. demonstrated that sensing systems must maintain a generalization capability with an F1-score above 97.1% on independent validation sets (unseen patients) to account for the vast inter-patient variability in gait characteristics [32]. In summary, technological evolution in recent years marks the entry of PD monitoring into a “bio-integrated” era, where sensing systems are no longer isolated sensors but complete closed loops that transform physical parameter capture into digital biomarkers by aligning with clinical imperatives.

To provide a comprehensive benchmarking of PD monitoring technologies, Table 1 synthesizes hardware performance metrics with clinical validation parameters.

### 2.2. Flexible Sensing Mechanisms and Advanced Material Systems

Having clarified the specific physical performance requirements of core motor symptoms in PD for sensing systems, this section delves into the underlying sensing mechanisms that support these benchmarks. The core value of flexible sensors lies in their ability to accurately convert subtle human mechanical deformations, pressure distributions, or bioelectrical signals into digitally processable electrical features through advanced functional materials and multi-scale microstructural designs. Current sensing technologies have evolved from early single-modality pressure sensing to a stage characterized by the parallel advancement of multi-physical field coupled sensing and neuroelectrophysiological monitoring. To achieve precise quantification of tremors and gait abnormalities in real-world scenarios, researchers have focused on developing devices based on diverse signal transduction principles, including piezoresistive, capacitive, ionic electric double-layer (EDL), self-powered, and electrophysiological mechanisms [18].

#### 2.2.1. Piezoresistive Sensors

Due to the high sensitivity, the simplified circuit design and low fabrication costs of piezoresistive sensors, they have occupied a central role in assessing motor functions—such as tremor and gait analysis—for patients with PD. However, conventional flexible piezoresistive sensors often face a challenging trade-off between achieving high sensitivity and maintaining a broad linear detection range.

To capture subtle tremor signals while simultaneously monitoring large-scale movements, researchers have introduced complex biomimetic microstructure designs. Inspired by the slit organs of spiders and the interlocking mechanisms of beetle wings, a hierarchical framework combining bio-inspired microcracks and interlocking structures has been developed. This architecture leverages exponential changes in contact area to achieve an ultra-low limit of detection (LOD) of 25 mg and a wide linear strain range of 0.2 to 80%, providing a robust physical foundation for precise identification of early PD tremors [27].

Beyond surface microstructures, mechanical metamaterial engineering has emerged as a novel strategy for modulating electromechanical performance. By incorporating chiral metamaterials with negative Poisson’s ratio (auxetic) properties or 3D cubic lattice structures with spherical voids, sensors can generate inward strain concentration effects under pressure. This structure-guided deformation not only significantly enhances piezoresistive sensitivity but also effectively mitigates the hysteresis phenomenon common in conventional flexible materials under large deformations, thereby enabling omnidirectional and accurate sensing of gait characteristics [37,38].

To meet the requirements of wearable comfort and cost-effectiveness for long-term health monitoring, research is increasingly focusing on green and accessible material systems. Low-cost material systems, such as carbon-ink functionalized textiles and paper-based touchpads, have demonstrated effectiveness in tremor and gait capture [39,40]. Furthermore, macro-structural modifications, such as the introduction of geometric slits, can further optimize bending sensitivity, better accommodating the monitoring needs of complex kinetic areas like finger joints [41].

#### 2.2.2. Capacitive and Ionic Sensors

Compared to piezoresistive sensors, capacitive sensors offer superior linearity, lower hysteresis, and exceptional static signal stability, which makes them highly effective for long-term gait monitoring and fine motor assessments. However, conventional capacitive sensors are often limited by extremely small initial capacitance and low sensitivity, making it difficult to accurately capture the subtle hand tremors characteristic of PD.

The ionic capacitive achieves a capacitance per unit area that far exceeds the magnitude of traditional dielectric layers. Inspired by the fingerprint structure of human skin, researchers have utilized starch gel electrodes coated with silver paste to construct micron-scale ridge patterns, significantly enhancing the rate of change in interfacial capacitance. These dextran-based flexible pressure sensors not only possess high sensitivity but also demonstrate excellent biocompatibility and biodegradability, providing an environmentally friendly, high-performance solution for home-based rehabilitation assessment in PD patients [35].

Environmental stability remains a significant challenge for wearable sensors. Conventional hydrogel sensors are prone to failure during long-term wear due to dehydration and drying; researchers have addressed this by introducing eutectogels as the sensing medium. By leveraging the low volatility and wide operating temperature range of Deep Eutectic Solvents (DES) combined with an acrylic/acrylamide matrix, eutectogel sensors have demonstrated exceptional stability during real-time monitoring of PD tremors. Integrated with machine learning, these systems can effectively classify PD symptoms and assess their severity, maintaining a high sensitivity factor (gauge factor) of 3.7 even under harsh environmental conditions [24].

Furthermore, structural engineering plays a critical role in enhancing capacitive sensing performance. 3D auxetic lattice metamaterials, fabricated via additive manufacturing, can serve as structural scaffolds for capacitive sensors. The inward collapse effect produced by this specific geometry under pressure allows for the precise regulation of electrode spacing and dielectric distribution, achieving structure-guided tactile perception. Compared to traditional positive Poisson’s ratio porous structures, this platform significantly improves sensor sensitivity for plantar pressure mapping [37]. Regarding fabrication processes, electrospun PVAc-graphene nanofiber dielectric layers endow sensors with excellent breathability and flexibility, enabling seamless integration into smart insoles for high-precision spatiotemporal gait analysis [33]. For monitoring specific tremor frequencies, flexible capacitive systems employing an AC bridge and a two-step calibration method can effectively filter out environmental interference, achieving clinical-grade quantitative analysis of Parkinsonian tremors [26].

#### 2.2.3. Self-Powered Sensors: Triboelectric and Piezoelectric Mechanisms

To satisfy the requirements for low-power or even zero-power consumption during 24/7 long-term monitoring of PD, self-powered sensing technologies have demonstrated immense potential. These sensors directly transduce mechanical energy from human movement into electrical signals, primarily through two mainstream modalities: TENG and Piezoelectric sensors [42].

In PD assessment, researchers have utilized flexible arm-mounted TENG sensors to capture the mechanical work generated by muscle movements, enabling the quantitative evaluation of rigidity and bradykinesia without the need for an external power supply [43]. To address the risk of mechanical damage in complex home environments, self-healing TENGs based on graphene oxide-polyacrylamide (GO-PAM) hydrogels have been proposed. This material not only exhibits extreme stretchability but also rapidly restores sensing performance after damage, ensuring the continuity of gait monitoring [44].

To address the challenge of capturing high-frequency tremors unique to PD patients, a novel sensor inspired by Kirigami structures and piezoelectric electret air gaps was developed. This architecture compresses the response time to 2.5 ms with a high dynamic sensitivity of 725 pC/N, enabling the distortion-free capture of tremor signals up to 20 Hz and providing exceptional temporal precision for clinical tremor subtyping [23].

Furthermore, advancements in manufacturing processes have accelerated the integration of self-powered systems. Piezoelectric PLA ferroelectret sensors, fabricated in situ via 3D printing technology, can be seamlessly integrated into personalized insoles to capture gait events in real-time during walking [45]. By employing hybrid piezoelectric-triboelectric designs, future wearable systems (such as smart socks) will not only monitor gait parameters but also harvest plantar impact energy to power small medical terminals, thereby constructing an integrated “sensing-collection-feedback” intelligent closed-loop ecosystem [13,46].

#### 2.2.4. Multimodal Sensing Integration

Single-modal sensors often encounter limitations when capturing the intricate and multifaceted clinical characteristics of PD. To achieve the transition from monitoring a “single pathological sign” to characterizing a “full-dimensional digital phenotype,” researchers are increasingly focusing on the development of integrated multimodal sensing systems.

The most prevalent integration strategy involves combining electrophysiological signals with kinematic parameters. For instance, by integrating sEMG with IMUs, a system can simultaneously monitor limb trajectories and perform in-depth analysis of muscle activation patterns and antagonistic muscle cocontraction. This multimodal fusion significantly enhances the robustness of tremor recognition and demonstrates remarkable foresight in predicting FoG, occasionally capturing physiological anomalies even before physical movement is obstructed [29,31]. In more sophisticated system designs, such as the flexible wireless platform developed by Hou, synchronized EEG, EMG, and ACC signals enable correlation analysis between the central nervous system and peripheral motor performance [25].

Another emerging trend is the hybrid integration of various physical sensing mechanisms. By incorporating flexible pressure arrays, bending sensors, and IMUs into a single wearable device—such as smart gloves or insoles—parameters including joint position, grip strength, and tremor frequency can be acquired concurrently [30]. Regarding plantar pressure monitoring, hybrid platforms (e.g., the “On-foot Gait Lab”) combining electrospun piezoelectric-capacitive sensors with IMUs can precisely distinguish between static load distribution and dynamic footprint characteristics. This provides multidimensional evidence for the clinical assessment of postural stability and the medication “ON-OFF” status in PD patients [33,47]. This paradigm shift from “discrete sensing” toward “integrated multimodal perception systems” not only improves diagnostic accuracy but also establishes the data foundation for future closed-loop precision interventions [48].

**Figure 2:**
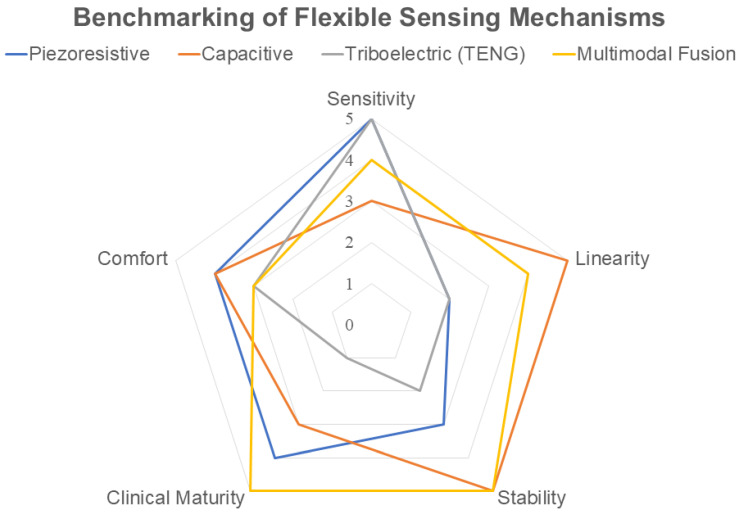
Quantitative benchmarking of mainstream flexible sensing mechanisms for PD monitoring. The scoring (0–5) represents a relative qualitative assessment, where 0 indicates low performance or suitability and 5 indicates excellent performance or high suitability in each dimension.

While Table 2 provides a detailed literature-based comparison, the multi-dimensional trade-offs between these sensing mechanisms are further visualized in the radar chart in Figure 2. By evaluating five critical dimensions—sensitivity, linearity, stability, clinical maturity, and comfort—this benchmarking explicitly demonstrates why multimodal fusion represents the most robust path for long-term clinical deployment compared to individual sensing modalities.

### 2.3. Flexible Bioelectronics for Non-Motor Symptom Monitoring

Beyond motor manifestations, PD encompasses a significant burden of non-motor symptoms (NMS), including sleep disturbances, cognitive impairment, and autonomic dysfunction. Flexible bioelectronics are uniquely suited for NMS monitoring due to their high skin-conformability, allowing for ‘imperceptible’ and continuous data collection that traditional rigid devices cannot achieve. For example, REM Sleep Behavior Disorder—a critical prodromal marker—can be monitored using ultra-thin e-skin patches that capture nocturnal respiratory patterns and subtle movements without disrupting the patient’s natural sleep cycle. Additionally, cognitive decline can be assessed through ‘digital proxies,’ such as gait variability and step-time fluctuations measured by flexible strain sensors, which reflect the executive function and cognitive load of the patient. By integrating these multidimensional signals, flexible sensing ecosystems enable a shift toward a holistic ‘digital phenotype’ of PD, providing clinicians with objective data across the full spectrum of the disease [49].

### 2.4. Advanced Manufacturing Processes and System Reliability Assessment

The transition of flexible sensors from laboratory prototypes to clinical applications hinges on balancing manufacturing precision, system integration, and long-term operational robustness, which constitutes the core challenge in evolving PD monitoring systems from hardware development to clinical deployment.

#### 2.4.1. Individualized Customization and Additive Manufacturing

For the highly personalized gait characteristics and anatomical differences of PD patients, additive manufacturing (3D printing) demonstrates high clinical versatility. Samarentsis et al. utilized dual-extrusion 3D printing for the monolithic integration of conductive TPU electrode plates and air-gap dielectric layers, enabling rapid customization of smart insoles tailored to individual foot shapes [28]. The device passed over 2280 cyclic tests and could quantify CoP trajectories in real-time, providing objective metrics for assessing fall risks associated with postural instability. Furthermore, Latsch et al. fused piezoelectric PLA sensors in situ within the insole structure, not only eliminating the common risk of interlayer delamination under long-term impact but also achieving sensing speeds superior to traditional force-sensitive resistors [45].

#### 2.4.2. Signal Integrity and Electromagnetic Environment Adaptability

In complex home monitoring scenarios, EMI is a major obstacle affecting the purity of sEMG and subtle tremor signals. Zhang et al. proposed dispersing liquid metal droplets in an elastic matrix to construct flexible films with excellent EMI shielding capabilities, ensuring that electrophysiological signals maintain clinical-grade diagnostic precision in environments full of electronic noise [29]. However, the performance loss brought by system integration cannot be ignored; research shows that the mechanical shunting effect of the packaging layer leads to a system-level sensitivity attenuation of approximately 2.1-fold compared to individual sensors, necessitating a precise trade-off between raw hardware performance and packaging robustness during the design phase.

#### 2.4.3. Long-Term Monitoring Robustness and Patient Adherence

Enhancing long-term patient adherence hinges on device durability and interfacial comfort. Addressing physical damage from prolonged wear, Park et al. developed sensing patches with self-healing capabilities using dynamic covalent bond networks, significantly extending device lifespan [50]. Additionally, modular sEMG leggings developed by Moore et al. employed waterproof encapsulation processes, ensuring no signal degradation after standard wash cycles, marking the transformation of monitoring devices from “experimental prototypes” to “daily life tools” [31]. At the skin-sensor interface, natural biogels developed by Lan et al. achieved stable adhesion while avoiding skin allergies, while the FLEXER textile electrodes by Lee et al. utilized lamellar protrusion structures to enhance contact stability and breathability, supporting 24/7 long-term PD monitoring and capturing complete circadian motor fluctuation cycles [20,51].

## 3. Artificial Intelligence-Driven Decision Support Systems: From Raw Sensing to Clinical Insights

### 3.1. Core Motor Feature Extraction and Signal Preprocessing

Efficient signal preprocessing and feature extraction constitute the cornerstone of building robust AI models capable of transforming flexible sensing data into clinical decision support. When capturing pathological signals in PD, flexible sensors frequently encounter challenges such as non-stationary environmental noise, baseline drift, and complex motion artifacts. Consequently, this section focuses on advanced signal processing techniques designed to extract digital biomarkers with clear clinical semantics.

**Figure 3:**
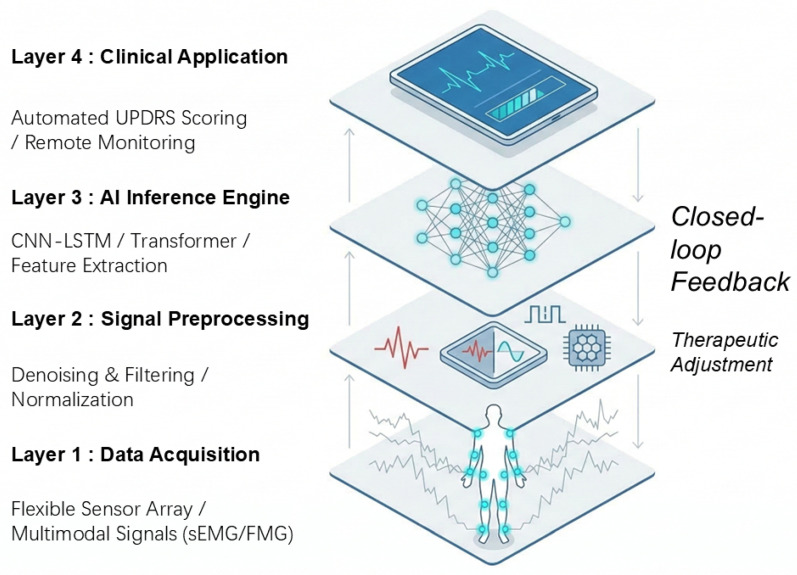
Hierarchical architecture and data workflow of the AI-enabled flexible sensing ecosystem. The framework highlights the progression from multimodal data acquisition (Layer 1, where blue circles denote sensor locations) and signal preprocessing (Layer 2, where red and blue lines represent raw and processed signals, respectively) to deep learning-based inference (Layer 3) and final clinical decision support (Layer 4).

The transition from raw physical stimulus to actionable clinical insights requires a well-structured computational pipeline. To provide a holistic view of this integration, Figure 3 illustrates the hierarchical architecture of the AI-enabled flexible sensing ecosystem. This workflow systematically organizes the process into four functional layers, emphasizing the closed-loop feedback necessary for personalized Parkinson’s disease management.

#### 3.1.1. Signal Cleaning and Artifact Suppression

The dynamic contact interface between flexible electronics and the skin is highly susceptible to motion artifacts. As Wu et al. mentioned, signals captured during long-term home monitoring exhibit high non-stationarity due to the diversity of patients’ activities of daily living (ADL) [10]. To address this, researchers commonly employ adaptive filtering or Empirical Mode Decomposition (EMD) to deconvolve pathological tremor signals from low-frequency interference caused by large-scale limb movements. Moore et al. further emphasized the necessity of applying power-line notch filtering (50 Hz) and band-pass filtering (20–450 Hz) to sEMG signals to ensure accurate capture of muscle cocontraction features during FoG prediction [31]. Additionally, Shama et al. and Chakraborty et al. demonstrated the advantages of Discrete Wavelet Transform (DWT) for multi-scale denoising, which effectively preserves the transient characteristics of tremor impulses while filtering out high-frequency random noise [52,53].

Furthermore, the long-term reliability of wearable flexible sensors is frequently compromised by environmental temperature fluctuations, which induce baseline drift and signal instability. While conventional filters address physiological artifacts, specialized compensation frameworks are required for environmental robustness. As demonstrated by Liu et al., integrating advanced temperature compensation algorithms—such as those based on optimal baseline selection and negative exponential amplitude compensation—is essential for ensuring signal fidelity and reducing false alarms in long-term monitoring scenarios [54].

#### 3.1.2. Digital Biomarkers and Feature Extraction

Feature extraction has evolved from traditional time-frequency domain analysis toward more sophisticated nonlinear dynamic features.

1. Time-Frequency Domain Features: Morinan et al. demonstrated the central role of Fast Fourier Transform (FFT) in tremor quantification [55]. By extracting the Power Spectral Density (PSD) energy ratio within the 4–6 Hz band, precise identification of resting tremors can be achieved [56,57]. Li et al. focused on the morphological features of time-domain waveforms, utilizing the inter-tap interval to quantify the rhythmicity and degree of bradykinesia during finger-tapping tasks [30].
2. Nonlinear Dynamic Features: Huo et al. suggested that relying solely on linear features is insufficient to describe the complexity of PD gait [58]. This study introduced Approximate Entropy (ApEn) and the Lyapunov Exponent to quantify gait stability and chaotic behavior. Such nonlinear analyses can sensitively capture subtle rhythmic fluctuations preceding FoG episodes, providing a digital basis for high-sensitivity early warning.

#### 3.1.3. Multimodal Fusion and Clinical Alignment

With increasing sensor integration, multimodal feature fusion has become key to enhancing model generalization [59]. Liu et al. indicated that integrating multidimensional features from accelerometers, gyroscopes, and flexible pressure sensors significantly improves the accuracy of early PD screening [34]. At the system integration level, Zhang et al. proposed a spatiotemporal alignment strategy to synchronize flexible plantar pressure mapping data with lower-limb sEMG signals, achieving complementary “kinetic-electrophysiological” representation at the feature level [29]. This multimodal fusion not only improves FoG detection accuracy but also ensures explicit clinical diagnostic significance through Pearson correlation analysis with MDS-UPDRS scales (Kappa = 0.833).

Furthermore, a bibliometric analysis by Qi et al. reveals that current research trends are shifting from “discrete feature extraction” to “end-to-end raw data input” [60]. This transition provides a richer information flow for the deep learning models discussed in the following sections, such as Convolutional Neural Networks (CNNs) and Transformers.

To maintain a clear boundary between current clinical feasibility and long-term research visions, we categorize the AI paradigms discussed in this review into three distinct tiers of maturity. Currently deployable technologies primarily consist of established deep learning models such as CNNs and Long Short-Term Memory (LSTM)s that have demonstrated high accuracy in specific motor symptom detection tasks within controlled environments. Emerging experimental systems include multimodal fusion frameworks and edge-AI implementations that are currently undergoing clinical trial validation to bridge the gap between laboratory and home settings. Finally, long-term conceptual AI frameworks, such as Large Multimodal Models and fully autonomous closed-loop AGI ecosystems, represent the future paradigm of Parkinson’s care. While these conceptual systems offer transformative potential for personalized medicine, they remain in the speculative stage and require significant advancements in data privacy, algorithmic interpretability, and clinical safety before they can be integrated into routine medical practice.

### 3.2. Deep Learning Models and Pathological State Classification

As the dimensionality of sensing data surges, traditional machine learning methods have gradually revealed limitations in processing the nonlinear, high-dimensional time-series signals generated by flexible sensors. Sigcha et al. and Ji et al. noted in a systematic review that deep learning (DL) architectures, with their end-to-end feature learning capabilities, have become the main paradigm for digital PD monitoring, with CNNs and RNNs being the most widely applied [61,62]. Current research is evolving from simple symptom classification to real-time detection with clinical early-warning value and lightweight deployment.

#### 3.2.1. Time-Series Modeling and Freezing of Gait Prediction

To address the temporal dependency of tremors and FoG in PD, researchers have extensively adopted RNNs and their variants. Al-Adhaileh et al. demonstrated through comparative analysis that LSTM networks outperform traditional models in capturing nonlinear gait features [11]. Based on this, Moore et al. utilized Gated Recurrent Units (GRUs) to model sEMG signals, not only achieving precise FoG detection but also capturing precursor signals of abnormal muscular cocontraction approximately 3 s before onset, thereby securing a critical window for real-time feedback intervention [31]. To further optimize computational efficiency, Yi and Hwang proposed a lightweight GRU model integrated with an Efficient Channel Attention (ECA) mechanism [63]. By enhancing temporal features across multi-sensor data, they achieved high-accuracy FoG monitoring on resource-constrained wearable devices.

#### 3.2.2. Spatio-Temporal Feature Fusion and Graph Convolutional Networks

For data with spatial distribution characteristics, such as plantar pressure mapping, CNNs have demonstrated exceptional representation capabilities. He et al. developed integrated sensing devices and utilized Graph Convolutional Networks (GCNs) to extract spatial correlations in fine motor finger movements, allowing early screening of PD [64]. To more meticulously analyze complex motor abnormalities, Huo et al. introduced a Multi-region attention Spatio-Temporal Directed Graph convolutional Network (Ma-ST-DGN) [58]. This model adaptively focuses on key regions in human skeletal kinematics, significantly enhancing sensitivity to subtle pathological signs by fusing global and local features. Furthermore, Liu et al. proposed an automated UPDRS gait scoring framework based on sensor fusion and deep learning, successfully mapping wearable data to clinical gold standards and providing decision support for precision remote medicine [34].

#### 3.2.3. Edge-Side Lightweight Models and Real-Time Monitoring Optimization

Driven by the stringent requirements for long battery life and low latency in flexible wearables, model compression has emerged as a research hotspot for 2025–2026. Ozkoc et al. developed a lightweight 1D CNN for tremor monitoring, which, through post-training 8-bit quantization and pruning techniques, compressed the model size by over 70% without significant loss in accuracy, enabling millisecond-level real-time inference on embedded platforms [65]. Borzì et al. further demonstrated an edge-computing-based FoG recognition framework [66]. By processing data locally, the system not only reduced communication latency associated with cloud transmission but also significantly enhanced patient data privacy. Additionally, Twala et al. emphasized the potential of ensemble learning and transfer learning to improve the robustness of AI models, showcasing superior generalization performance when addressing significant inter-patient variability within the elderly population with PD [67].

### 3.3. Edge Computing and the Telemedicine Ecosystem

**Figure 4:**
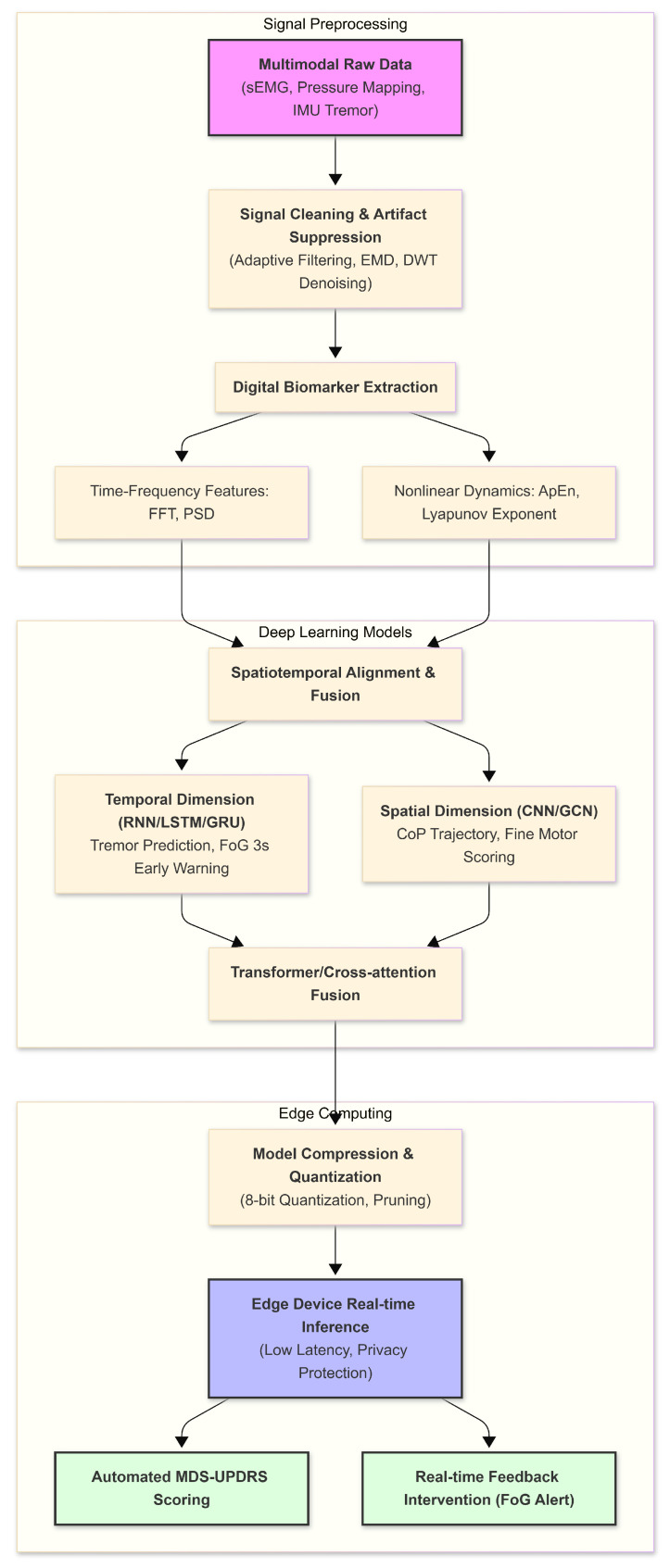
Flowchart of the AI-driven signal processing and deep learning pipeline. The architecture spans from raw data preprocessing to spatiotemporal feature fusion and automated clinical scoring.

Integrating high-performance AI algorithms into flexible wearable devices to construct an “edge-cloud” collaborative telemedicine ecosystem is fundamental to the long-term home management of PD. Due to the highly paroxysmal nature of PD symptoms such as FoG, traditional cloud-based processing models encounter bottlenecks including data transmission latency, potential privacy breaches, and high power consumption. Consequently, the intervention of edge computing technology enables the instantaneous processing of pathological features at the sensor terminal, thereby significantly enhancing the timeliness of clinical interventions. Figure 4 provides a detailed flowchart of the AI-driven signal processing and deep learning pipeline, mapping the entire trajectory from raw data cleaning and spatiotemporal feature fusion to automated clinical scoring.

To address the constraints of edge-side computational power, Chen et al. developed a lightweight detection network named SMV-FoG [32]. By optimizing the network topology, this research reduced inference latency to 177.31 ms while maintaining a high recognition rate of 97.1%, providing technical assurance for the real-time triggering of rescue biofeedback interventions. Simultaneously, Ozkoc et al. achieved ultra-lightweight deployment of tremor detection algorithms using model pruning and quantization [65]. Their study indicated that a substantial reduction in model parameters could nearly double the battery life of embedded systems with minimal precision loss, which is of critical significance for home scenarios requiring 24/7 continuous monitoring.

At the systemic level of the telemedicine ecosystem, Borzì et al. proposed an edge-based gait recognition architecture, emphasizing the central role of local data processing in safeguarding patient privacy [66]. The system automatically filters out irrelevant ADL and synchronizes only critical pathological assessment metrics to cloud-based physician workstations, enabling efficient remote follow-up. Furthermore, Sigcha et al. pointed out in their systematic review that future PD monitoring systems will evolve into a closed-loop ecosystem: flexible sensors capture subtle signs, edge algorithms quantify severity in real-time, and cloud platforms generate digital reports to dynamically adjust medication dosages or neurostimulation parameters based on assessment results [61]. Such an edge-computing-based ecosystem not only breaks geographic barriers to high-quality medical resources but also provides an integrated solution from “monitoring” to “decision-making” for the precision rehabilitation of PD patients.

## 4. Clinical Application Status, Cross-Domain Fusion, and System-Level Challenges

With the maturation of flexible sensing technologies and AI algorithms, the management of PD is shifting from a “hospital-centered” to a “home-based full-course” paradigm. This chapter explores the clinical consistency of sensor technology with gold standards, the cross-domain fusion of multimodal sensing, and the systemic challenges encountered in real-world medical deployment.

### 4.1. Clinical Validation and Benchmarking

The clinical value of the massive digital data streams generated by flexible sensors hinges on their ability to accurately map to the MDS-UPDRS, the globally recognized medical scoring system. Despite the high sampling frequency and sensitivity of these sensors, transforming resistance and charge fluctuations into interpretable clinical metrics remains a primary research focus.

#### 4.1.1. Automated Scoring and Clinical Gold Standard Alignment

Achieving automated UPDRS scoring is essential for alleviating clinician burden and enhancing assessment objectivity. Liu et al. developed a gait scoring framework based on flexible sensor fusion and deep learning [34]. By extracting kinematic parameters such as stride length, walking speed, and gait variability, their study utilized CNNs to achieve high consistency with MDS-UPDRS gait sub-items. The results indicate that sensor-based automated scoring not only replicates clinical visual assessments but also provides higher granularity data on disease evolution by capturing “micro-waveform” features, such as subtle shifts in plantar pressure.

However, it is crucial to note that high correlation with clinical ratings like MDS-UPDRS does not necessarily imply functional equivalence. While wearables capture ‘micro-waveform’ features, the relationship between these digital signals and patient-perceived disability or therapeutic decision-making requires further validation in large-scale multicenter trials to ensure true clinical meaningfulness.

#### 4.1.2. Simplified Scales and Computational Search in Remote Monitoring

In long-term home monitoring, complete MDS-UPDRS assessments are difficult to execute frequently due to their complexity. Morinan et al. utilized an exhaustive computational search to identify a simplified set of MDS-UPDRS metrics suitable for remote monitoring [55]. This study proved that monitoring specific key movements (e.g., finger tapping, sit-to-stand transitions) allows for effective tracking of daily fluctuations in PD patients without losing diagnostic sensitivity. Furthermore, Wu et al. proposed an explainable machine learning model that automatically identifies the “ON/OFF” medication states of patients [10]. The agreement between their scoring results and clinical diaries significantly outperformed traditional self-reporting modes, providing empirical evidence for precision medication adjustment.

#### 4.1.3. Standardization of Digital Biomarkers

Digital biomarkers are increasingly becoming novel endpoint metrics in clinical trials. A bibliometric analysis by Qi et al. indicated that parameters such as gait entropy and dominant tremor frequency distribution have been widely accepted as objective standards for evaluating neurodegenerative progression [60]. However, standardization across different devices and algorithms remains a hurdle. Twala emphasized in their AI-driven precision diagnosis research that establishing cross-platform open-source feature databases—such as common feature extraction protocols based on diverse flexible materials—is an essential path for the large-scale deployment of digital PD healthcare [67].

### 4.2. From Physical to Biochemical Multimodal Sensing

While kinematic-based flexible sensors have made significant progress in capturing the external physical manifestations of PD, they primarily monitor the “consequences” of disease progression rather than the underlying “causes,” namely the fluctuations in neurotransmitter concentrations like dopamine [68,69]. Therefore, integrating physical motion sensing with non-invasive biochemical monitoring to construct multimodal fusion systems is the most forward-looking trajectory in flexible electronics for 2025–2026 [36].

#### 4.2.1. Neurotransmitter Monitoring in Sweat and Tears

Non-invasive analysis of biofluids (e.g., sweat, tears) offers a new window for real-time molecular diagnosis of PD. Qi et al. noted that electrochemical sensors integrated on flexible substrates can detect dopamine and cortisol levels in sweat with high sensitivity [60]. These biochemical sensors utilize MXene or gold-nanoparticle-modified electrodes to enhance electron transfer rates, achieving nanomolar (nM) limits of detection [36,70]. Compared to pure gait monitoring, biochemical markers more directly reflect the degree of damage to dopaminergic neurons in the substantia nigra, providing physiological evidence for early PD screening.

#### 4.2.2. Pharmacodynamic Assessment and Dyskinesia Monitoring

Long-term levodopa treatment often leads to significant motor fluctuations (ON/OFF phenomena) and levodopa-induced dyskinesia (LID). Moreau et al. proposed the “levodopa-paradox,” emphasizing the complexity of quantifying motor fluctuations during pharmacological intervention [1]. Through physical-chemical dual-mode sensors, the systems can simultaneously record biochemical changes and limb tremor frequencies before and after peak blood drug concentrations. Kim et al. demonstrated an electromagnetic MXene-mediated platform that not only perceives muscle micro-tremors caused by medication fluctuations but also monitors physiological charge changes induced by drug metabolism, offering neurologists precise recommendations for medication timing windows [36].

#### 4.2.3. Spatiotemporal Fusion of Multimodal Data

Achieving deep fusion of physical and biochemical signals faces challenges of data asynchrony and disparate scales. Recent research tends to utilize Transformer architectures (such as the lightweight model proposed by Yi in 2025) to process these heterogeneous data streams [63]. By performing cross-attention between kinematic features in physical coordinates and concentration gradients from electrochemical sensors, AI models can identify specific “digital phenotypes,” enabling early warnings through subtle biochemical abnormalities before significant motor degradation occurs.

### 4.3. Closed-Loop “Sense-and-Treat” Frameworks

The ultimate goal of sensing technology is not merely observation but achieving automated closed-loop management from perception to intervention. For PD patients, delivering physical or physiological stimuli at the instant of FoG or severe tremor onset holds profound clinical significance [48,71]. It is essential to distinguish wearable-based monitoring systems from fully implantable closed-loop Deep Brain Stimulation (DBS) devices. While implantable DBS offers direct neural modulation, it involves significant surgical risks and costs. In contrast, the flexible wearable ecosystems discussed in this review prioritize non-invasive, long-term monitoring, serving as a complementary ‘digital companion’ to optimize pharmacological therapy, rather than a direct replacement for invasive neuromodulation.

#### 4.3.1. Instantaneous Feedback Intervention for FoG

When AI algorithms predict an impending FoG episode, the system can immediately trigger interventions via flexible actuators. Borzì et al. demonstrated a closed-loop framework based on edge computing that combines tactile cues [66]. By applying specific frequency vibration stimuli to the patient’s ankle, the system helps break motor rigidity and significantly reduces fall risks. The latency of the “detection-response” in this closed-loop system was compressed to less than 200 ms using Chen’s SMV-FoG model, essentially reaching the level of human-like reflexes [32].

#### 4.3.2. Neural Reprogramming and Precision Electrical Stimulation

Regarding deeper interventions, Kim et al. proposed a disruptive technological path: utilizing flexible MXene-based platforms for neural reprogramming therapy [36]. Upon detection of specific neurodegenerative electrical signals, the system can emit specific waveforms via in situ microelectrodes to induce functional repair of damaged dopaminergic neurons. Integrating high-sensitivity sensing and in situ treatment into a single flexible patch marks a new phase of intelligent bioelectronic therapy for PD management.

### 4.4. System-Level Challenges in Real-World Settings

Despite the excellent performance of flexible sensing and AI in laboratory settings, transitioning these into clinically viable medical-grade products requires overcoming system-level challenges ranging from data security to patient adherence—the “Valley of Death” between prototypes and commercialization.

#### 4.4.1. Data Privacy and Ethics

Motor data and physiological signals from PD patients constitute highly sensitive personal privacy [20]. During long-term monitoring, data transmission and storage in the cloud face risks of interception or misuse. Borzì et al. pointed out that adopting edge computing architectures is not only for reducing latency but also for performing critical feature extraction locally, achieving a “data-stays-on-device” privacy protection mode [66]. Furthermore, Twala emphasized the importance of “Responsible ML,” ensuring that algorithmic decision-making is transparent and unbiased to avoid misdiagnosis across different genders or age groups due to imbalanced training data [67].

#### 4.4.2. Adherence and Wearability for Long-Term Monitoring

For patients with neurodegenerative diseases, device comfort directly determines the continuity of monitored data. Currently, the breathability, skin-friendliness, and washability of flexible sensors remain physical bottlenecks for large-scale applications. Sigcha et al. noted in a systematic review that many patients discontinue use due to skin irritation or cumbersome operational procedures [61]. Future research should explore highly breathable porous nanofiber substrates (e.g., via electrospinning) and incorporate self-healing materials to withstand mechanical wear during daily activities.

#### 4.4.3. Data Scarcity and Generalization

The high cost of obtaining quality labeled clinical data leads to a “small-sample” dilemma for deep learning models. Twala noted that due to significant individual differences in tremor frequency and gait habits, models trained on single datasets often fail to generalize to complex real-world populations [67]. Utilizing generative AI for synthetic data and applying transfer learning from general human activity recognition models to PD-specific tasks are effective paths to mitigate this issue.

#### 4.4.4. Power Management and Low-Power Architectures

The lightweight design of flexible sensors limits battery capacity. While model compression techniques proposed by Ozkoc et al. reduce inference power, high-frequency sampling and wireless transmission remain energy-intensive [65]. Future system integration must consider deep coupling of high-efficiency energy harvesting (e.g., TENG) with ultra-low-power edge computing units to achieve device “self-sufficiency” or ultra-long battery life.

## 5. Conclusions and Outlook

With the intensification of global population aging, the precision monitoring and early intervention of PD have emerged as major challenges in public health. This review has systematically retraced the full-chain evolution of flexible sensing technologies in PD management: from the bio-inspired design and mechanistic innovation of underlying materials (Section 2), to breakthroughs in AI algorithms for feature extraction, model compression, and real-time edge prediction (Section 3), and finally to clinical explorations of multimodal biochemical fusion and integrated “sense-and-treat” systems (Section 4).

### 5.1. Summary of Core Achievements

Currently, flexible monitoring systems have evolved from initial “physical quantity capture” into intelligent platforms capable of “clinical semantic understanding”:

1. Hardware Level: Through the introduction of advanced materials such as MXene, liquid metal, and self-healing hydrogels, sensors have achieved a critical balance between sensitivity and mechanical robustness, satisfying the hardware requirements for long-term continuous monitoring.
2. Software Level: Deep learning models—such as the GRU architecture by Moore and the lightweight Transformer by Yi—have successfully pushed the early warning window for FoG to 3 s prior to onset. Furthermore, by leveraging edge computing technologies (e.g., Chen), inference latency has been compressed to millisecond levels, addressing the real-time processing bottlenecks of wearable devices [31,32,63].

### 5.2. Future Evolution Trajectories and Predictions

Looking ahead 3–5 years, the field of flexible PD monitoring is expected to be shaped by three core trends:

1. Deep Integration of AGI and Large Multimodal Models (LMMs): Future systems will transcend single-task classification. Instead, they will utilize AGI or medical-specific large models to perform semantic interpretation of long-term, multidimensional sensing data (kinematic, electrophysiological, biochemical, and acoustic). AI will directly generate diagnostically interpretable clinical recommendations rather than merely providing numerical scores [72,73,74,75].
2. Integrated “Sense-and-Treat” Synergetic Systems of Invasive and Wearable Devices: Building upon the neural reprogramming concept proposed by Kim, future closed-loop systems will feature synergy between flexible skin-surface patches and miniaturized implantable stimulators [36]. When wearable sensors capture specific pathological signatures, they will trigger precise Deep Brain Stimulation (DBS) or micro-adjustments of drug pumps via synchronized internal-external data, achieving true “precision dosing.”
3. Standardized Global Digital Biomarker Repositories and Federated Learning: To overcome the small-sample data bottleneck (a challenge noted by Twala in 2025), standardized global sensor protocols will gradually be established [67]. Through Federated Learning, models across different institutions can be collaboratively trained without sharing raw patient data, thereby constructing highly generalizable “Digital Parkinsonian Portraits” for universal precision monitoring across diverse ethnicities and age groups.

### 5.3. Barriers to Clinical Translation

Despite the rapid technological evolution of flexible sensing ecosystems, several critical barriers still hinder their transition from laboratory prototypes to clinical-grade medical products. A primary challenge remains the significant translational gap, where high-performance individual sensors often fail to maintain system-level reliability and robustness in real-world, long-term monitoring scenarios. As highlighted in our comparative analysis, many current studies are limited to small cohort sizes in controlled laboratory environments, lacking the large-scale validation required to ensure efficacy across diverse patient populations. Furthermore, the absence of standardized digital biomarker protocols and the stringent requirements for medical device regulatory compliance pose significant hurdles for broad commercialization. Addressing these barriers—through multicenter clinical trials and the development of more durable, motion-artifact-resistant hardware—is essential to move beyond proof-of-concept demonstrations and achieve the full potential of digital Parkinson’s disease healthcare.

### 5.4. Final Conclusions

In summary, flexible wearable technology is leading the paradigm shift in Parkinson’s disease assessment from “intermittent clinical snapshots” to “real-world, full-course interactions.” Although technical barriers remain in data privacy, biocompatibility, and long-term power management, the interdisciplinary fusion of materials science, microelectronics, and generative AI will eventually position flexible monitoring systems at the core of closed-loop PD management, significantly improving the quality of life for millions of patients worldwide.

## Figures and Tables

**Table 1:** Comprehensive Benchmarking of PD Symptom Monitoring: From Hardware Requirements to Clinical Validation.

Symptom	Clinical Endpoint	Key Sensor Requirements & Metrics	*n*	Environment	Rep. Ref. & Performance
Resting Tremor	Freq. (4–6 Hz) & Amplitude (MDS-UPDRS III)	LOD < 50 mg; ms-level response; High SNR.	1	Lab (Healthy)	Guo [27]: 25 mg LOD.
			3	Lab (Healthy)	Xie [23]: 2.5 ms response.
			-	Lab (Bench)	Khan [26]: pF resolution.
Bradykinesia	Tapping speed, range, and rhythmicity.	Linearity ($R^{2}>0.99$); IMU + sEMG multi-modal fusion.	40	Clinical Lab	Li [30]: Kappa 0.833.
		High-fidelity signal capture; low hysteresis.	28	Clinical Lab	Zhang [29]: Multi-modal.
Gait/FoG	Prediction lead time (>3 s); FoG frequency.	Electrophysiological precursor capture; Low latency (<200 ms).	10	Controlled	Moore [31]: sEMG capture.
		Wide linear range (MPa); Dynamic pressure mapping.	177	Database	Chen [32]: 97.1% F1-score.
Postural Instability	COP migration; Gait variability.	Pressure range > 800 kPa; Precise COP tracking.	-	Lab (Healthy)	Onorati [33]: Hybrid platform.
		High durability (>10,000 cycles); Baseline stability.	21	Clinic/Home	Liu [34]: Scoring accuracy.
Rigidity & NMS	Muscle stiffness; Sleep (RBD); Cognition.	Bio-compatibility; Low Young’s modulus; Self-healing.	1	Lab (Healthy)	Zheng [35]: Biodegradable.
		High SNR; Sensitivity to subtle physiological changes.	-	Animal Model	Kim [36]: MXene-mediated.

Notes: *n*, cohort size; LOD, limit of detection; COP, center of pressure; FoG, freezing of gait; NMS, non-motor symptoms; RBD, REM sleep behavior disorder.

**Table 2:** Critical Technical Comparison of Sensing Mechanisms and Their Translational Barriers.

Mechanism	Material Strategy	Primary Advantage	Critical Limitation	Ref.
Piezoresistive	Microcrack/Auxetic AMMs	Ultra-high sensitivity for capturing subtle resting tremors.	Significant hysteresis and non-linearity during dynamic movements.	[27,37]
Capacitive	Ionic/Eutectogel	High linearity and baseline stability; excellent biocompatibility.	Parasitic capacitance interference and lower sensitivity ranges.	[24,39]
Piezoelectret	Kirigami/Porous Gaps	Fast dynamic response (2.5 ms); ideal for high-frequency vibration.	Vulnerable to environmental humidity and long-term charge decay.	[23]
Triboelectric	Hydrogel/TENG	Self-powered capability; reduces system standby power consumption.	Signal instability caused by sweat and moisture interference.	[43,44]
Multimodal	IMU + sEMG + Array	Highest diagnostic precision via motor-muscle data correlation.	High computational load and complex data fusion requirements.	[30,34]

## Data Availability

No new data were created or analyzed in this study. Data sharing is not applicable to this article.
